# Life‐Prolonging Treatment Preferences and Their Association With Health Care Utilization and End‐Of‐Life Experiences in Older Adults

**DOI:** 10.1111/jgs.70055

**Published:** 2025-08-29

**Authors:** Lesli E. Skolarus, Chun Chieh Lin, Sara Hassani, Ran Bi, James F. Burke

**Affiliations:** ^1^ Department of Neurology Northwestern University Feinberg School of Medicine Chicago Illinois USA; ^2^ Department of Neurology Ohio State University Columbus Ohio USA

**Keywords:** end of life, older adults, preferences

## Abstract

**Background:**

People markedly differ in their preferences for life‐prolonging treatments (LPT). We explored the association between LPT preferences and healthcare utilization and end‐of‐life (EOL) experiences.

**Methods:**

We conducted a retrospective cohort study using data from 5373 older adults in the National Health and Aging Trends Study (NHATS) linked to Medicare/Medicaid. Among them, 1564 died, and 1124 had proxies who completed the Last Month of Life module. We categorized LPT preferences (accept or reject) in severe disability or pain scenarios, and we assessed healthcare utilization (hospitalization, ICU visits, receipt of LPT, hospice), total cost, and days out of home in the last year of life, place of death, and EOL experiences.

**Results:**

The average age of respondents was 76.7 years (SD 5.9), 55.1% female, and 7.8% Black people. Sixty‐nine percent would reject all LPT; 5.3% would accept LPT only in the severe disability scenario; 15.8% would accept LPT only in the pain scenario; and 9.9% in both. There was no association between LPT preferences and hospitalization, ICU visits, receipt of LPT, or costs. Respondents accepting LPT for pain spent more time away from home (3.4 days, 95% CI: 0.3, 6.6, *p* = 0.03). Among decedents, preferences were not linked to receipt of LPT, hospice use, or EOL care quality. Those accepting LPT for severe disability and pain had higher odds of dying in a hospital (OR = 1.8, 95% CI 1.1–3, *p* < 0.03) compared to those who would reject LPT.

**Conclusions:**

LPT preferences established in hypothetical scenarios were not associated with health care utilization, cost, or perceived quality of end‐of‐life. As LPT preferences should influence care, subsequent work, such as understanding preference measurement, real‐world preference changes, or the limited opportunity for preferences to influence care, is warranted.

**Summary**

- Key points
  ○ Preferences for LPT were not associated with receipt of LPT, health care utilization, or perceived quality of end of life.
  ○ Preferences to accept LPT in the setting of severe disability and pain were associated with higher odds of death in the hospital.
- Why does this paper matter?
  ○ As preferences for care should influence receipt of care, subsequent work, should explore other measures of treatment preferences such as documented clinical discussions and the mechanism by which preferences for care influence receipt of care and end of life experiences.

## 1 Introduction

Patient involvement in medical decision‐making is a core component of care [1, 2]. As the population ages and the number of people with chronic disease increases [3], planning for the medical care people want to receive may become more important. In addition, improved experience for dying individuals is a high national priority among the public, providers, medical societies, healthcare organizations, and policymakers [4].

Advance care planning (ACP) is a process that supports people in understanding and sharing their personal values, life goals, and preferences to prepare for medical care decisions [5]. One objective of ACP, by capturing people's preferences in ACP documents or conversations, is to narrow the gap between the care desired and care received; ensuring that people receive care aligned with their values and preferences.

We sought to understand the relationship between preferences for life‐prolonging treatment (LPT) and healthcare utilization. We also explored the association between LPT preferences and proxy's perception of the quality of end‐of‐life (EOL) among decedents. We hypothesized that older adults who would accept LPT would have higher rates of healthcare utilization, receipt of LPT, and worse quality of EOL than those who would reject LPT.

## 2 Methods

### 2.1 Data Source and Study Populations

The study used data from the National Health and Aging Trends Study (NHATS)‐linked to the Medicare/Medicaid dataset, including Medicare Fee‐for‐Service claims, Medicare Advantage claims, and Medicaid MAX and TAF claims. NHATS is a nationally representative sample of Medicare beneficiaries aged 65 and older. NHATS oversampled those over the age of 80 and Black individuals [6]. Participants undergo annual in‐person interviews. In the 2012 wave of NHATS, a random 1/3 of the sample (*N* = 2000) received a module about EOL plans and care, and this module was repeated in the 2018 wave across the entire NHATS sample (*N* = 5547). The module included two hypothetical scenarios asking older adults to imagine they were at the end of their lives and had a serious illness. They would need to choose whether to “receive” or “stop/reject” LPT in specific EOL situations. These situations involved being in “constant, severe physical pain” with preserved cognition and function (pain scenario) or being unable to “speak, walk, or recognize others” but without pain (severe disability scenario). We identified 5373 respondents (i.e., older adults) with non‐missing responses to at least 1 LPT preference question in the 2012 wave (*N* = 1702) or the 2018 wave (*N* = 3632). We tracked respondents from the base wave up to the latest available year as repeated observations within respondents to assess healthcare utilization for each follow‐up year. Among them, 1564 NHATS participants later died (i.e., decedents). Finally, proxies—typically surviving family members‐ of a subset of these decedents (*n* = 1124) completed the NHATS Last Month of Life Module. This study was approved by the Institutional Review Board of the University of Michigan and the Ohio State University.

### 2.2 Outcomes

Healthcare utilization was quantified using the linked NHATS‐Medicare/Medicaid dataset. Mortality was assessed through the NHATS case status, the Medicare Beneficiary Summary File (MBSF), the Medicaid MAX Personal Summary File, and the Medicaid TAF Demographic and Eligibility File. Hospitalizations were identified using Medicare and Medicaid inpatient claims. Intensive Care Unit (ICU) stays were determined based on revenue codes (020 and 0210) and ICU indicators in the inpatient claims. Life‐prolonging treatments were identified using ICD‐9‐CM/ICD‐10‐CM procedure codes from inpatient claims, which included procedures such as gastrostomy, tracheostomy, intubation/MV, hemodialysis, cardiopulmonary resuscitation (CPR), and nutritional support [7]. The total number of days spent away from home for each follow‐up year was calculated based on hospitalization days, days in skilled nursing homes, and days in long‐term care facilities. Hospitalization days were tracked using Medicare and Medicaid inpatient claims. Days in skilled nursing homes were counted through Medicare skilled nursing home claims, while days in long‐term care facilities were tracked using Medicaid long‐term care claims. The total medical cost was calculated as the sum of the amounts paid by Medicare and/or Medicaid insurance and out‐of‐pocket expenses paid by the patient. These costs were log‐transformed to account for skewed data.

Among decedents, we identified hospice use, receipt of LPT, and costs in the last year of life. Hospice utilization was identified through Medicare Hospice claims, as well as Medicaid MAX and TAF Other Services Files. The number of LPTs received was counted in the last year of each decedent's life. The total cost in the last year of life was calculated by summing the amounts paid by the insurance and the patient in the last year of life from all medical claims and then log‐transformed to account for skewed data.

Among decedents who had proxies who completed the last month of life module, we evaluated the place of death, perception of the quality of EOL care, and perception of the quality of EOL [8]. The place of death was classified as home/non‐hospital hospice versus other locations (see supplemental text for further details on measures).

### 2.3 Primary Exposure

Our primary exposure was the NHATS respondent's LPT preference in pain and severe disability scenarios—reject all LPT, accept LPT in the severe disability scenario only, accept LPT in the pain scenario only, and accept LPT in both severe disability and pain scenarios. Most respondents answered their LPT preferences only in one NHATS wave, while around one‐tenth (pain scenario: *N* = 606, severe disability scenario: *N* = 628) participated in both the 2012 and 2018 wave EOL modules. Respondents who participated in both waves were more likely to be White, reject LPT, and have dementia [9]. For the analyses related to healthcare utilization, we used the respondents' initial LPT preferences. To examine the association between LPT preferences and decedent outcomes and proxy‐reported decedents' EOL experiences in the last month of life, we referred to the LPT preferences closest to the time of their death.

### 2.4 Covariates

We identified participant demographic characteristics through their base wave response (age group at base wave, sex, race/ethnicity [non‐Hispanic White, Black, Hispanic, other, or missing], marital status [married/living with a partner vs. others], and education), and health characteristics (self‐reported comorbid conditions [myocardial infarction, congestive heart failure, hypertension, arthritis, osteoporosis, diabetes, lung, stroke, cancer], dementia, depression, and anxiety). Dementia was identified as the NHATS' probable dementia, which has been validated [10]. Depression and anxiety were measured by the Patient Health Questionnaire (PHQ‐2) and the Generalized Anxiety Disorder (GAD‐2) questionnaires. Original entitlement to Medicare was determined through the MBSF file, based on old age vs. disabled/ESRD. Medicare‐Medicaid dual eligibility was identified through the MBSF file in the base wave year. We also included preference expression, which refers to whether individuals have expressed their preferences for medical treatment in the event of serious illness. This includes having a durable power of attorney for health care, a living will, or speaking to someone about their medical treatment preferences. The analysis also includes the base wave year (2012 vs. 2018). For the model examining the place of death, nursing home residence in the previous round was included as a covariate.

### 2.5 Statistical Analysis

We conducted descriptive analyses to characterize the study population by their LPT preference category. Regression models were specified based on the distribution and scale of the outcome variable. We used Poisson regression models to examine the association between LPT preferences and the number of hospitalizations, ICU visits, and receipt of LPT while adjusting for respondents' sociodemographics, comorbidities, and expression of preferences. Additionally, we used linear regression models to estimate log‐transformed total costs and the number of days spent away from home across LPT preferences. Given that our analyses involved multiple observations from the same respondent, we applied round‐specific weights and clustered the data by each respondent to account for the NHATS sample design. We also conducted sensitivity analyses by treating the cohort as a convenience sample and employed multi‐level analyses that clustered data by respondents without applying survey weights.

To evaluate the association between LPT preferences and healthcare utilization in the last year of the NHATS decedent's life, we performed a logistic regression to estimate the likelihood of utilizing hospice services, a Poisson regression to estimate the number of LPTs received in the last year of life, and a linear regression to estimate log‐transformed total healthcare costs in the last year of life across different LPT preferences.

To evaluate the association between LPT preferences and EOL experiences among the NHATS decedents, we performed logistic regressions to assess the association between LPT preferences and place of death, perceived quality of EOL care, and perceived quality of EOL. We conducted linear regressions to estimate symptoms, unmet needs, and insufficient interaction with the care team across different LPT preferences, adjusting for the decedent's characteristics and expression of preferences. We addressed missing data using multiple imputations and conducted sensitivity analyses. We applied analytic weights from the base wave for these cross‐sectional non‐recurrent event analyses.

For all analyses, we performed sensitivity analyses repeating the models incorporating interaction terms of LPT preference and preference expression (no LPT Preference‐no Preference Expressed, no LPT Preference‐Preference Expressed, LPT Preference‐no Preference Expressed, LPT Preference‐Preference Expressed). All statistical analyses were conducted using SAS 9.4 (SAS Institute Inc., Carry, NC) and STATA 18 (STATA Corp., College Station, TX).

## 3 Results

### 3.1 Patient Characteristics of the Older Adult Cohort

Among the 5373 older adults, 69% would reject all LPT, 5.3% would accept LPT in the severe disability scenario only, 15.8% would accept LPT in the pain scenario only, and 9.9% would accept LPT in both scenarios. Overall, respondents were followed for 1 to 10 years after completing the LPT survey. On average, the respondents were 76.7 (SD 5.9) years old, 55.1% were female, 7.8% were Black, 52.9% were married, and 57.7% had an education level greater than high school. Respondents who would reject LPT in both scenarios were older, had higher levels of education, were less likely to be Black or Medicare‐Medicaid dual eligible, and had lower anxiety levels than those in the other LPT groups (Table 1).

**TABLE 1:** Characteristics, utilization, and healthcare cost among older adults.

	Total	No LPT	LPT in severe disability	LPT in pain	LPT in severe disability and pain	*p*
	*N* = 5373	3664 (69)	295 (5.3)	787 (15.8)	627 (9.9)	
	*N* (weighted col.%)					
Ever express EOL	4116 (76.7)	2961 (80)	212 (74.9)	593 (75.6)	350 (56.1)	< 0.01
Age (mean, SD)	76.7 (5.9)	77.1 (7)	76.3 (6.7)	75.7 (5.9)	75.8 (7.2)	< 0.01
Female	3077 (55.1)	2121 (56.1)	161 (52.6)	466 (55.3)	329 (49)	0.09
Black	1094 (7.8)	566 (5.8)	96 (13.4)	169 (8.1)	263 (18.8)	< 0.01
Married	2465 (52.9)	1666 (51.7)	148 (58.5)	392 (57.6)	259 (50.4)	0.02
Education						< 0.01
< High school	1124 (17.1)	693 (15.6)	73 (19.2)	147 (15.8)	211 (28.7)	
High School	1401 (25.1)	991 (26.2)	81 (26.5)	188 (22.6)	141 (20.8)	
> High school	2848 (57.7)	1980 (58.1)	141 (54.3)	452 (61.6)	275 (50.6)	
Round						0.73
2	1702 (24.7)	1138 (24.8)	91 (22.4)	250 (24.2)	223 (26)	
8	3671 (75.3)	2526 (75.2)	204 (77.6)	537 (75.8)	404 (74)	
Entitle						0.09
Due to age	4921 (91.6)	3395 (92.2)	265 (91)	717 (91.4)	544 (88.1)	
Due to disable	452 (8.4)	269 (7.9)	30 (9)	70 (8.7)	83 (11.9)	
Part D	3960 (73.7)	2667 (72.4)	224 (76.6)	585 (75.7)	484 (78)	0.06
Dual eligible beneficiaries	861 (13.2)	485 (10.9)	67 (18.8)	120 (12.4)	189 (27.8)	< 0.01
Comorbidity						
Myocardial infarction	903 (14.9)	620 (15)	60 (18)	117 (12.6)	106 (16.9)	0.16
Congestive heart failure	1208 (21)	823 (20.3)	63 (16.3)	171 (18.6)	151 (22.2)	0.27
Hypertension	3889 (68.3)	2632 (68.5)	221 (69.7)	557 (65.9)	479 (69.7)	0.61
Arthritis	3607 (64.7)	2459 (64.8)	189 (63.3)	538 (65.4)	421 (63.2)	0.90
Osteoporosis	1542 (28.6)	1068 (28.5)	66 (23.5)	235 (29.5)	173 (30.4)	0.39
Diabetes	1531 (27.3)	989 (26.8)	110 (33.8)	231 (26.8)	201 (28.4)	0.20
Lung	1097 (20.1)	759 (20.4)	53 (18)	156 (19.9)	129 (19.3)	0.86
Stroke	652 (10.1)	446 (10.3)	33 (7.6)	87 (9.7)	86 (10.7)	0.54
Cancer	1679 (30.3)	1216 (32.4)	94 (31.1)	212 (25.5)	157 (22.7)	< 0.01
Physical incapacity (mean, SD)	2.7 (3)	2.7 (2.9)	3.0 (3.2)	2.3 (2.6)	3.3 (3.7)	< 0.01
Depression	592 (9.3)	401 (9.1)	47 (11.5)	59 (6)	85 (14.4)	< 0.01
Anxiety	528 (8.8)	350 (8.7)	34 (8.9)	65 (7.1)	79 (12.4)	0.04
Dementia						< 0.01
No	4628 (90.1)	3188 (90.6)	235 (86.3)	706 (93)	499 (83.6)	
Possible	542 (7.7)	365 (7.6)	42 (9.9)	55 (5.4)	80 (11.4)	
Probable	203 (2.2)	111 (1.8)	18 (3.8)	26 (1.6)	48 (5.1)	
	Weighted Mean (SD)					
Hospitalizations	0.3 (0.7)	0.3 (0.7)	0.3 (0.8)	0.2 (0.7)	0.3 (0.9)	0.15
ICU visits	0.1 (0.4)	0.1 (0.4)	0.1 (0.4)	0.1 (0.3)	0.1 (0.5)	0.22
Life prolonging treatments	0.03 (0.3)	0.03 (0.3)	0.03 (0.2)	0.03 (0.2)	0.04 (0.4)	0.35
Log transformed total estimated costs	7.2 (2.6)	7.2 (2.5)	7.2 (2.7)	7.2 (2.4)	7.2 (3.1)	0.99
Total estimated costs	12571.4 (153434.8)	10771.7 (31572.6)	11660.4 (29946.9)	11558.4 (46932.5)	27304.3 (523175.9)	0.58
Non‐home days—hospital/SNF/LTC	7.6 (34.1)	7.1 (31.8)	9.6 (39.8)	9.3 (40.2)	7.2 (34.6)	0.41

Abbreviations: ICU = intensive care unit; LTC = long‐term care; SNF = skilled nursing facility.

### 3.2 Association of LPT Preferences With Healthcare Utilization and Cost

On average, respondents were followed up 1632 (SD 943) days after the base wave interview. In that time‐frame, older adults experienced an average of 0.3 (SD 0.7) hospitalizations per year, 0.1 (SD 0.4) ICU stays, received 0.03 (SD 0.3) LPTs, spent 7.6 (SD 34.1) days away from home, and had a total of $12,571.4 (SD $153,434.8) in healthcare costs. Relatively small differences were observed across LPT preferences (Table 1).

After adjusting for respondents' demographics, comorbidities, and expression of preferences, acceptance of LPT was not associated with a higher number of hospitalizations, ICU visits, receipt of LPT, or cost than those who would reject any LPT (Table 2). Respondents who would accept LPT for pain were more likely to spend extended time away from home (3.4, 95% CI: 0.3, 6.6, *p* = 0.03) than those who would reject LPT.

**TABLE 2:** Associations between LPT preferences and healthcare utilization and cost among older adults^a^.

	Hospitalizations	ICU visits	Life prolonging treatments	Total estimated costs (log transformed)	Not home days—hospital/SNF/LTC
	P.E. (95% CI)	P.E. (95% CI)	P.E. (95% CI)	P.E. (95% CI)	P.E. (95% CI)
LPT					
No LPT	0	0	0	0	0
LPT in severe disability	0.1 (−0.1–0.3)	0.2 (−0.1–0.5)	−0.02 (−0.6–0.6)	−0.1 (−0.4–0.3)	2.1 (−1.4–5.5)
LPT in pain	−0.1 (−0.2–0.1)	−0.03 (−0.3–0.2)	0.2 (−0.3–0.7)	0.1 (−0.1–0.3)	3.4 (0.3–6.6)*
LPT in severe disability and pain	−0.05 (−0.2–0.1)	−0.03 (−0.4–0.3)	0.1 (−0.3–0.6)	−0.1 (−0.4–0.2)	−2.1 (−4.1–0.02)
Expression	0.01 (−0.1–0.1)	−0.02 (−0.2–0.2)	−0.1 (−0.4–0.3)	0.3 (0.1–0.5)**	−0.7 (−2.9–1.4)

*Note*: *p < 0.05, **p < 0.01.

Abbreviations: ICU = intensive care unit; LTC = long‐term care; SNF = skilled nursing facility.

^a^ Adjusted for sociodemographics, comorbidities, and expression of preferences.

Respondents who would accept LPT in all scenarios had total costs for each follow‐up year that were twice as high as those who would reject LPT (LPT in all scenarios: $27,304.3 vs. no LPT: $10,771.7). After adjusting for respondents' sociodemographic and comorbidities, there was no significant difference in total healthcare costs across LPT preferences: for severe disability, Coef −0.06 (95% CI: −0.4, 0.3); for pain, Coef 0.07 (95% CI: −0.1, 0.3); and for pain and severe disability, Coef −0.07 (95% CI: −0.4, 0.2) (*p* > 0.1). Respondents who expressed their preferences for medical treatment incurred higher total medical costs than those who did not (Coef 0.3 [95% CI: 0.1, 0.5], *p* < 0.01).

### 3.3 Decedent Cohort

Overall, one‐fifth of the respondents (*N* = 1564, weighted 21.4%) died during the study period. Of these, 53.7% utilized hospice services, and on average, the decedents received 0.2 (SD 0.8) LPT in the last year of their life (Table S1). There were small but not statistically significant trends in hospice utilization and receipt of LPT. Compared to decedents with preferences to accept LPT in both scenarios, decedents who would reject LPT in both scenarios had greater hospice utilization (54.8% vs. 44%, *p* = 0.11 across all LPT groups, Table S1) and received fewer LPTs (0.2 vs. 0.3, *p* = 0.62 across all LPT groups). After adjusting for the decedent's socioeconomic status, comorbidities, and preference expression, there was no significant association between preferences for LPT and use of hospice services, receipt of LPT, and costs in the last year of life (Table 3).

**TABLE 3:** Associations between LPT preferences and hospice, total cost of the last year of life, LPT received in the last year of life among NHATS decedents^a^.

	Hospice utilization	Log‐transformed total cost of the last year of life	# Life‐prolong treatment received in the last year of life
	OR (95% CI)	P.E. (95% CI)	P.E. (95% CI)
LPT			
No LPT	1	0	0
LPT in severe disability	0.8 (0.5–1.4)	0.2 (−0.2–0.6)	0.08 (−0.7–0.9)
LPT in pain	1.2 (0.8–1.8)	−0.1 (−0.5–0.2)	−0.2 (−0.8–0.5)
LPT in severe disability and pain	0.8 (0.5–1.2)	−0.02 (−0.4–0.3)	0.2 (−0.3–0.7)
Expression	1.1 (0.8–1.6)	0.2 (−0.06–0.5)	−0.2 (−0.6–0.3)

^a^ Adjusted for sociodemographics, comorbidities, and expression of preferences.

### 3.4 Proxy Perception of Decedents' EOL

Among NHATS decedents, 1124 had proxies who completed the NHATS Last Month of Life Module; 73.4% would reject all LPT, 6.8% accepted LPT in the severe disability scenario only, 11.6% accepted LPT in the pain scenario only, and 8.2% accepted LPT in both scenarios.

On average, nearly half (49.1%) of NHATS decedents died at home or in a non‐hospital hospice setting (Table S2). After adjusting for decedents' characteristics and expression of preferences, there was no statistically significant association between LPT preferences, quality of EOL care, and quality of EOL (Table 4). Preferences to accept LPT in the setting of severe disability and pain were associated with higher odds of death in the hospital (OR = 1.8, 95% CI 1.1–3, *p* < 0.03). Our findings were substantively similar when the predictor was changed to the LPT preference and expression interaction.

**TABLE 4:** Associations between LPT preferences and place of death, quality of end‐of‐life, and quality of end‐of‐life care among NHATS decedents with proxies who completed the last month of life module**.

	Quality of end‐of‐life care summary	Place of death at home/non‐hospital hospice	Place of death at hospital	Alertness	Ability to get out of bed	Sum of symptoms	Sum of unmet needs	Sum of insufficient interaction with care team
	OR (95% CI)	OR (95% CI)	OR (95% CI)	OR (95% CI)	OR (95% CI)	P.E. (95% CI)	P.E. (95% CI)	P.E. (95% CI)
LPT								
No LPT	1	1	1	1	1	0	0	0
LPT in severe disability	1.3 (0.7–2.5)	1.1 (0.6–2.1)	1.3 (0.6–2.6)	0.8 (0.5–1.6)	1 (0.5–1.9)	0.1 (−0.1–0.3)	−0.1 (−0.9–0.6)	−0.3 (−0.8–0.2)
LPT in pain	1.6 (1–2.5)	0.9 (0.5–1.4)	1.4 (0.8–2.5)	0.9 (0.6–1.4)	0.9 (0.6–1.5)	0.03 (−0.1–0.1)	0.3 (−0.2–0.7)	−0.2 (−0.6–0.2)
LPT in severe disability and pain	1.3 (0.8–2.3)	0.7 (0.4–1.1)	1.8 (1.1–3)*	0.6 (0.4–1.1)	0.7 (0.4–1.2)	−0.1 (−0.2–0.1)	−0.3 (−1.0–0.5)	−0.1 (−0.6–0.3)
Expression	1.3 (0.9–2)	1.2 (0.8–1.8)	0.9 (0.6–1.4)	1.3 (0.9–1.8)	1 (0.7–1.5)	0.1 (0.01–0.2)*	0.1 (−0.3–0.5)	−0.2 (−0.5–0.1)

*Note*: *p < 0.05, **adjusted for sociodemographics, comorbidities, and expression of preferences.

## 4 Discussion

In this national sample of community‐dwelling older adults, we found rare, if any, relationship between hypothetical LPT preferences and healthcare utilization, cost, or end‐of‐life experience. Specifically, we found that LPT preferences were not associated with healthcare utilization or cost among older adults. Respondents who would accept LPT in the setting of pain were more likely to spend time away from home than those who would reject LPT. Among decedents, LPT preferences were not associated with receipt of LPT, hospice utilization, or cost. In the last month of life, LPT preferences were not associated with quality of EOL care, quality of EOL, or cost. Preferences to accept LPT in the setting of severe disability and pain were associated with a higher likelihood of in‐hospital death.

Several explanations for the limited association between preferences and the outcomes explored may exist. First, the influence of patient preferences may be small compared to other patient factors (i.e., disease severity) [11]. On average, decedents had 0.7 ICU visits and received 0.2 LPTs in the last year of their life, suggesting that there may have been little opportunity (i.e., sudden death from cardiac arrest, subarachnoid hemorrhage) for preferences to guide EOL care. Second, the quality of care and outcomes can be influenced by local providers, hospitals, or health systems. Prior research suggests that the intensity of EOL care is associated with hospitals and local medical resources (e.g., hospital bed supply and medical specialists per capita) and regional systemic factors specific to individual hospital–physician networks [12, 13]. For example, Barnato et al. found regional variation in EOL treatment intensity is unlikely due to variation in patient preferences because preferences were similar across regions, suggesting that provider, hospital, or regional factors may be associated with the variation [13]. Our study adds to this work by showing that LPT hypothetical preferences were not associated with the actual receipt of LPT. Given the complexity underlying EOL care pathways and interactions, future models may need to examine multi‐level correlates of preferences and utilization together.

Second, LPT preferences were assessed using hypothetical scenarios, which may not be valid measures of actual “real‐world” preferences. While consistent with common approaches to elicit preferences [13], such assessments may not reflect true preferences in specific clinical situations or circumstances involving uncertainty or tradeoffs. Future studies could consider other options to measure preferences, such as a review of clinical documentation, which includes the interpersonal context of patients' discussions with healthcare providers, which might influence their decisions. Further, while we found that preferences were stable across time when older adults are healthy or living with chronic disease overall percent of older adults providing the same LPT response in between wave 2 and 8 was 76% (95% CI: 72%–79%) for pain scenario and 86% (95% CI: 83%–89%) for severe disability scenario [9], preferences may change at the moment when people face a serious illness or at the EOL [14, 15].

While a strength of our study is that it is one of the most representative assessments to date on the preferences for care across patients with various illnesses, prior work has supported the value of distinguishing between different trajectories of dying [16, 17]. For example, family‐reported end‐of‐life care quality has been shown to be significantly better for cancer and dementia patients than for patients with ESRD, cardiopulmonary failure, or frailty [18].

Our results are aligned with a recent study that found that ACP billing was not associated with LPT, treatment limitation, or death among patients admitted to the hospital [19]. However, other studies limited to decedents found an association with ACP billing and receipt of lower overall end‐of‐life treatment intensity and reduced odds of hospitalizations, emergency department visits, and ICU stays within 1 month of death, as well as lower likelihood of dying in the hospital [20, 21]. Among decedents, we found that preferences to accept LPT were associated with death while in a hospital. Combined, these findings suggest that preferences for care expressed closer to death may have a greater impact on care and outcomes than hypothetical preferences.

Our study has several limitations. We could be underpowered to find important associations. Overall, receipt of LPTs was rare. We hypothesized that preferences for LPT would be a proxy for other healthcare measures, such as ICU visits and hospice utilization. However, some respondents who did not accept LPT may still have expressed a preference for intensive EOL treatment, for example, accepting ICU admission while rejecting LPT. Our findings are limited to older community‐dwelling adults who completed the survey; individuals residing in long‐term or nursing care facilities are not represented. Of note, Black Americans comprise 9% of the US population over the age of 65 compared to 7.8% included in our study [22]. Black people are more likely to accept LPT than White people, but given the small amount of underrepresentation, this is unlikely to substantially impact the findings [9].

In conclusion, LPT preferences, as assessed in hypothetical scenarios conducted years prior, were not significantly associated with healthcare utilization, cost, or end‐of‐life experience. Our results should not change current clinical practice but encourage further study of LPT preferences. If confirmed, our unexpected findings would suggest that care for older adults may not always align with their preferences, raising important questions about the extent to which older adults' preferences meaningfully influence healthcare utilization, outcomes, and costs. Subsequent work should explore other measures of treatment preferences, such as documented clinical discussions and the mechanism by which preferences for care influence receipt of care and end‐of‐life experiences.

## Author Contributions

Study concept and design: L.E.S., J.F.B. Statistical analysis: C.C.L., R.B. Interpretation of data: L.E.S., J.F.B., C.C.L., R.B. Drafting of the manuscript: L.E.S., C.C.L., S.H. Critical revision of the article: L.E.S., C.C.L., R.B., S.H., J.F.B.

## Disclosure

The authors have nothing to report.

## Conflicts of Interest

The authors declare no conflicts of interest.

## Supporting information


**Data S1:** jgs70055‐sup‐0001‐Supinfo.pdf.
