# Supplementary material for: Life‐Prolonging Treatment Preferences and Their Association With Health Care Utilization and End‐Of‐Life Experiences in Older Adults
Source: J Am Geriatr Soc. 2025 Aug 29;73(10):3138–45. doi: 10.1111/jgs.70055 (PMC12554839; doi:10.1111/jgs.70055)
Supplement: Supplementary file 1 — Data S1: jgs70055‐sup‐0001‐Supinfo.pdf. [file JGS-73-3138-s001.pdf]

Supplement:

Supplemental text:

The quality of the EOL care was determined by the proxy's perception of care in the last month of the decedent's life, categorized as Excellent vs. Very Good/Good/Fair/Poor. The quality of the last month of life was evaluated using a scale that included alertness, the ability to get out of bed, symptoms, unmet needs, and interaction with the care team. Symptoms experienced by the NHATS decedents were counted based on whether they experienced pain, had any feelings of anxiety or sadness, and had trouble breathing (yes- 1, no- 0) (range 0-3). Unmet need was defined as whether the decedent had unmet needs for pain management, dyspnea management, anxiety/sadness management, and spiritual support (range 0-4). Insufficient interaction with the care team was counted by assessing whether the decedent was treated with respect (always vs. others), whether any decisions made about care conflicted with what the decedent would not have wanted (yes vs. no), whether care decisions were made without enough input from the decedent or their family (yes vs. no), and how often was informed about the decedent's condition (always vs. others) (range 0-4).

Supplemental Table 1: Healthcare utilization and costs among NHATS decedents

	total	no LPT	LPT in severe disability only	LPT in pain only	LPT in severe disability and pain	
	n=1564	n=1079 (71.1)	n=92 (5.9)	n=201 (13.1)	n=192 (9.9)	p value
			(n, weighted col %)			
Ever EOL Express	1195 (76.9)	872 (80.1)	144 (71.8)	144 (71.8)	114 (62.0)	<0.01
Age at death, Mean (SD)	80.61 (7.9)	80.88 (8.0)	81.13 (6.1)	79.75 (6.9)	79.5 (8.7)	0.11
Female	855 (52.9)	604 (55.2)	45 (46.3)	113 (50.9)	93 (43.2)	0.08
Black	338 (8.4)	175 (6.1)	25 (12.0)	54 (11.0)	84 (19.7)	<0.01
Education						<0.01
<high school	455 (25.4)	285 (22.7)	27 (24.5)	63 (31.7)	80 (37.5)	
high school	411 (27.0)	303 (28.6)	25 (30.4)	41 (19.8)	42 (23.4)	
>high school	698 (47.6)	491 (48.8)	40 (45.2)	97 (48.5)	70 (39.1)	
Married	592 (43.3)	398 (40.6)	42 (53.1)	83 (49.7)	69 (48.1)	0.05
Physical Incapacity (Mean, SD)	4.62 (4.0)	4.58 (4.0)	4.88 (4.0)	4.39 (3.8)	5.04 (4.9)	0.59
Depression	254 (15.7)	179 (16.2)	20 (13.3)	28 (12.7)	27 (17.5)	0.60
Anxiety	214 (14.4)	147 (14.0)	10 (8.0)	26 (15.0)	31 (20.4)	0.19
Probable/Possible Dementia	397 (21.3)	262 (20.2)	35 (38.3)	44 (18.6)	56 (23.2)	<0.01
Ever Had Comorbidity						
heart attack	374 (24.1)	266 (24.0)	26 (30.4)	40 (22.0)	42 (25.1)	0.59
heart disease	482 (31.6)	339 (31.8)	29 (30.7)	56 (28.8)	58 (34.9)	0.79
high blood press	1205 (75.3)	836 (77.0)	74 (70.6)	146 (68.7)	149 (74.4)	0.20
arthritis	1051 (65.6)	728 (66.4)	57 (64.5)	133 (61.7)	133 (66.2)	0.75
osteoporosis	446 (29.2)	310 (28.7)	23 (25.5)	65 (35.9)	48 (26.7)	0.31
diabetes	521 (34.9)	323 (32.2)	39 (45.5)	83 (41.4)	76 (39.5)	0.03
lung disease	393 (26.9)	283 (28.3)	23 (22.2)	44 (22.2)	43 (25.9)	0.38
stroke	297 (18.5)	214 (19.5)	11 (9.7)	33 (17.6)	39 (18.2)	0.26
cancer	570 (38.3)	411 (39.3)	38 (48.6)	73 (40.1)	48 (23.1)	<0.01
Entitle due to disability	161 (11.2)	100 (9.8)	7 (7.9)	26 (16.6)	28 (16.3)	0.06
Dual eligible	330 (19.8)	199 (18.3)	20 (19.6)	46 (20.1)	65 (30.8)	0.02
HMO	570 (35.0)	397 (34.8)	36 (39.3)	66 (30.1)	71 (40.1)	0.37
Medicare PartD	1119 (70.9)	764 (69.9)	67 (74.3)	139 (70.1)	149 (77.8)	0.33
Hospice Utilization	854 (53.7)	600 (54.8)	46 (48.2)	118 (57.9)	90 (44.0)	0.11

	weighted mean (sd)					
# Life Prolonging Treatments in last year	0.22 (0.9)	0.2 (0.8)	0.24 (0.8)	0.22 (0.8)	0.31 (0.9)	0.62
# ICU visits in last year	0.72 (1.2)	0.73 (1.1)	0.93 (1.1)	0.63 (1.1)	0.7 (1.4)	0.35
Log-transformed Last year of cost	9.9 (2.0)	9.9 (2.0)	10.1 (1.9)	9.9 (2.0)	9.8 (2.5)	0.73

19

Supplemental Table 2. Characteristics of the NHATS decedents who had proxies who completed the Last Month of Life Module Cohort

	Total	no LPT	LPT in severe disability only	LPT in pain only	LPT in severe disability and pain	
	N=1124	797 (73.37)	74 (6.84)	131 (11.62)	122 (8.17)	p value
	N (weighted col %)					
Ever Express EOL	879 (77.9)	652 (80.4)	54 (78.1)	98 (73.3)	75 (62.2)	0.01
Age at death (Mean, SD)	84.1 (8)	84.2 (8.1)	84.4 (6)	83.4 (7.4)	83.2 (9.3)	0.57
Female	622 (52.9)	453 (56)	37 (44.6)	69 (44.7)	63 (43.6)	0.04
Black	226 (7.8)	115 (5.6)	18 (10.5)	36 (10.4)	57 (22.4)	<0.01
Education						<0.01
<High school	312 (24)	194 (20.5)	22 (23.9)	46 (39.2)	50 (34)	
High school	298 (26.7)	226 (28.6)	19 (26.8)	28 (19.8)	25 (19.8)	
>High school	514 (49.2)	377 (50.9)	33 (49.3)	57 (41)	47 (46.2)	
Married	420 (43.3)	293 (40.7)	33 (56.6)	54 (50.6)	40 (45.6)	0.07
Physical Incapacity (Mean, SD)	4.8 (4.1)	4.8 (4.1)	5 (3.8)	4.7 (3.7)	4.7 (5)	0.97
Depression	190 (16.2)	139 (17.6)	19 (15.6)	21 (13.3)	11 (8.1)	0.11
Anxiety	158 (14.4)	107 (13.6)	13 (12.6)	20 (19.5)	18 (16.1)	0.50
Probable/Possible Dementia	397 (31.9)	259 (29.2)	33 (38.2)	53 (43.6)	52 (34.5)	0.03
Ever Had Comorbidity						
Heart attack	333 (29.8)	233 (28.5)	27 (38.1)	39 (32.2)	34 (30.2)	0.53
Heart disease	457 (41.2)	320 (40.4)	31 (42.6)	57 (43.8)	49 (44)	0.89
High blood press	914 (80.2)	644 (80.5)	62 (79.2)	104 (75.6)	104 (84.6)	0.59
Arthritis	849 (73.8)	599 (73.7)	55 (78.9)	98 (72.7)	97 (71.8)	0.82
Osteoporosis	390 (35.2)	276 (34.3)	24 (31.3)	51 (46)	39 (31.8)	0.16
Diabetes	412 (39.2)	264 (36.1)	32 (45.7)	61 (48.5)	55 (48.8)	0.04
Lung disease	370 (34.2)	265 (35)	22 (27)	44 (36.1)	39 (30.6)	0.61
Stroke	301 (25.2)	215 (25.6)	15 (15.9)	36 (28.9)	35 (24.6)	0.34
Cancer	500 (46.8)	360 (46.7)	39 (59.1)	63 (50.4)	38 (32.2)	0.03
Nursing Home in Previous Round	111 (9.1)	78 (9.5)	9 (8)	15 (10.4)	9 (4.2)	0.39
Place of Death						0.07
Home/non-hospital hospice	530 (49.1)	383 (49.9)	34 (52.8)	62 (48.7)	51 (39.4)	
Hospital/ambulance to hospital	332 (30)	216 (27.6)	26 (32.3)	42 (33.2)	48 (45.4)	
Nursing home/somewhere else	262 (20.9)	198 (22.6)	14 (14.9)	27 (18.1)	23 (15.2)	
Entitle due to disability	104 (10.7)	62 (8.8)	6 (8.4)	19 (21.4)	17 (14.7)	0.01
Dual eligible	236 (19.2)	147 (18.3)	15 (15.5)	30 (18.9)	44 (31.2)	0.08
HMO	421 (36.2)	301 (36.1)	29 (39.7)	46 (35)	45 (36.3)	0.95
Part D	817 (72.5)	575 (71.8)	51 (68.3)	74 (95)	96 (79.3)	0.49
Place of Death - home or non-hospital hospice	530 (49.1)	383 (49.9)	34 (52.8)	62 (48.7)	51 (39.4)	0.44
Quality of end-of-life care - excellent	505 (44.7)	355 (43)	33 (50.4)	64 (51.6)	53 (45.4)	0.55
Alertness - not everyday	532 (45)	378 (45.8)	37 (43.8)	63 (46)	54 (38.2)	0.66
Get out of bed - not everyday	697 (60.2)	494 (61.3)	48 (59.1)	81 (59.1)	74 (53.1)	0.72
Place of Death - Hospital	332 (30)	216 (27.6)	26 (32.3)	42 (33.2)	48 (45.4)	0.04
	Weighted Mean (SD)					
Symptoms Index	2.0 (1)	2.0 (1)	2.1 (1)	2.0 (1)	1.8 (1.3)	0.34
Unmet Needs Index	0.2 (0.6)	0.2 (0.5)	0.2 (0.5)	0.4 (0.9)	0.2 (0.6)	0.7
Insufficient Interaction with Care Team Index	0.5 (0.9)	0.6 (0.9)	0.4 (0.7)	0.4 (0.8)	0.5 (1)	0.17
